# The Veterinarian

**Published:** 1831-08

**Authors:** 


### BIBLIOGRAPHICAL NOTICE.

*The Veterinarian:* published monthly. From the commencement to July 1831. Edited by Messrs. PERCIVALL and YOUATT. Longman and Co.

WE cannot but feel interested and gratified by the rapid improvements which practitioners of veterinary medicine are making in their art, both on account of the vast importance of the duties which they have to perform, and because it is impossible that a decided progress can be effected in comparative anatomy, physiology, or pathology, without much benefit being derived to medical science in general. Many of the histories of disease, as they occur in the horse and other animals, which are contained in this very useful periodical, will be found extremely interesting to all medical readers, although they are especially addressed to the veterinary surgeon: nor can the lectures on the anatomy of the horse, which are given in the "Veterinarian," be either a dull or unprofitable study. The series of papers on Canine Madness, by one of the editors, Mr. YOUATT, which have since been published in a separate form, contain much valuable and important information: they comprise by far the best account with which we are acquainted, of the symptoms, post-mortem appearances, nature, origin, and preventive and curative treatment of rabies in the dog and other domestic animals.

The "Veterinarian" also contains many other excellent communications from Messrs. YOUATT, PERCIVALL, SEWELL, TURNER, FIELD, GOODWIN, &c. &c.

Until very recently, the members of the veterinary profession, in this country at least, have been but little known for their scientific acquirements, or even as well-grounded and judicious practitioners in their department of the healing art; but a very different estimate is now to be formed of them. It would be invidious to select particular individuals for eulogium, when so many deserve our warmest applause for their professional zeal and intelligence. We cannot refer to a better or more satisfactory source than the "Veterinarian," in proof of the extensive information and great practical ability possessed by many veterinary surgeons. Veterinary medicine is at length assuming the rank to which it is so well entitled: most of the individuals who now practise it are totally different from the "horse-doctor" of former times, both in character and professional talent. A veterinary medical society has been long established, and is attended by some of the most respectable practitioners. The discussions, which are reported in the "Veterinarian," evince an earnest anxiety to improve their art, by the reciprocal communication of interesting cases and improved modes of practice.

A course of Veterinary Lectures has also been delivered at the London University, by Mr. Youatt: they are correctly reported in the "Veterinarian," and we earnestly recommend them to students; they do infinite credit to the ability of the lecturer, both on account of the abundance of practical information which they contain, and of the perspicuous manner in which his opinions are communicated. In the introductory lecture, Mr. Youatt dwelt very forcibly upon the vast importance of the art of veterinary medicine: we agree with him, that "it is closely allied to, or is rather a part and portion of, medical science, and identified with the agricultural interests and most valuable resources of the country."

We must conclude our brief notice of "the Veterinarian," by expressing our most sincere wishes for its success; which is not, indeed, to be doubted, so long as it continues under the direction of Messrs. Percivall and Youatt, and is supported by such a host of intelligent contributors. We shall, from time to time, extract, from the subsequent numbers, any articles that we think likely to interest our readers.

### LITERARY INTELLIGENCE.

Mr. Waller has in the press a second edition of his "Elements of Practical Midwifery," which contains an additional quantity of matter, and will be published by S. Highley, Fleet street, in a few days.

Mr. Charles Severn will shortly publish "First Lines of the Practice of Midwifery, with Remarks on Fœticide, Infanticide, and the Forensic Evidence requisite in proof of the commission of those crimes."

